# Contact dermatitis to methylisothiazolinone*

**DOI:** 10.1590/abd1806-4841.20153992

**Published:** 2015

**Authors:** Maria Antonieta Rios Scherrer, Vanessa Barreto Rocha, Ana Regina Coelho Andrade

**Affiliations:** 1 Universidade Federal de Minas Gerais (UFMG) - Belo Horizonte (MG), Brazil

**Keywords:** Additives in cosmetics, Dermatitis, allergic contact, Preservatives, pharmaceutical

## Abstract

Methylisothiazolinone (MI) is a preservative found in cosmetic and industrial products. Contact dermatitis caused by either methylchloroisothiazolinone/methylisothiazolinone (MCI/MI or Kathon CG) or MI has shown increasing frequency. The latter is preferably detected through epicutaneous testing with aqueous MI 2000 ppm, which is not included in the Brazilian standard tray. We describe a series of 23 patients tested using it and our standard tray. A case with negative reaction to MCI/MI and positive to MI is emphasized.

Methylchloroisothiazolinone and methylisothiazolinone (MCI/MI), also known as Kathon CG or Euxyl K 100, has been used (in a fixed 3:1 mixture) as a preservative since the 70s. In 2000, MI started being commercialized alone for use in industrial products such as paints and adhesives, and since 2005 it has also been used in cosmetic products at concentrations ranging from 50 to 100ppm. Due to this extensive use, the number of allergy cases caused by MCI/MI or by MI alone has been increasing.^1-5^

Some examples of cosmetic products that contain MI are: products for children and babies, bath products, makeup (eyeliners, makeup removers, blush and face powder), hair care products (such as dyes and bleaches), nail and waxing products, moisturizing creams, sunscreen and baby wipes. According to the Food and Drug Administration, in 2010, about 2408 American cosmetic products used MI as a preservative.

Examples of occupational products that contain MI are: paints, glues, lacquers, cutting oils and other products for industrial use. So far there are neither rules establishing the maximum concentration allowed for use in finished products, nor a mandatory requirement to specify the MI content on product labels, which makes it difficult to identify this component in occupational products.

With regard to household cleaning products that contain MI, we may cite: dishwasher soaps, washing powders, laundry detergents, stain removers, fabric softeners, glass cleaners, products for wood protection and even the so-called "green" cleaning products.^3^

Although the epicutaneous testing with MCI/MI can only detect contact allergy to MI alone, about 40% of cases fail to be diagnosed, possibly due to its low concentration in this combination.^1,2,3,4,5^

Currently, MCI/MI is tested in Europe and in the USA in concentrations of 0.01% in aqueous solution (100ppm) and, in Brazil, in concentrations of 0.5% in petrolatum.^5^

Recent studies recommend MI concentrations of 2000 ppm in aqueous solution since at this concentration, more allergy cases are detected. Moreover there are no reports of sensitization by patch testing and chemical investigations have shown that MI is evenly distributed in aqueous solution, but not in petrolatum.^1,2,3^

MI sensitive patients also react to MCI, although the contrary is not necessarily true.^4,5^

MI is not tested in European, North American and Brazilian standard trays, which limits the possibility of making the diagnosis of MI allergies. Recent articles recommend its inclusion in European and North American trays.^4^

We studied a series of 23 patients tested between March and July 2014 with the Brazilian standard tray, which contains MCI/MI (FDA Allergenic) adding MI 0.2% in aqueous solution (Chemotechnique) to it. The tests were carried out by using the Finn Chamber patch test technique (EpitestLtd Or, Tuusula, Finland) on Scanpor tape (AS NorgeplasterAlpharma, Vennesla, Norway). Readings were taken at 48 and 96 hours, following the international reading standard. Allergic reactions were graded as +, ++ or +++ based on the intensity of positive reactions, which manifested as erythematous papules, vesicles and dissemination of the reaction with crusting and ulceration, respectively. The cosmetic tray (FDA Allergenic) and other complementary allergens were included in the investigation of some patients (when relevant).

The mean age of all 23 patients was 48.43 years (standard deviation of 14.95 years). 21 (91.30%) patients were women. 14 subjects (61%) showed a negative reaction to MCI/MI and MI; 8 subjects (35%) showed a positive reaction to MCI/MI and MI; and only one subject (4%) showed a positive reaction to MI and a negative reaction to MCI/MI.

Among those patients who showed a positive reaction to MI, 90% had disseminated lesions, being 90% on the legs, 60% on the trunk and abdomen, 50% on the hands and scalp, and 40% on the face. These data are consistent with a previous study on MCI/MI, except for the higher frequency of lesions on the face found in the current study.^5^

Among the aforementioned cases, we highlight the one that was positive for MI and negative for MCI/MI. A 27-year-old caucasian female teacher was referred for patch testing due to a 2-year-old history of itchy and recurrent rash on the legs, abdomen, trunk and face (Figures 1 and 2). She had a previous history of bronchitis. The patient was tested with the standard and the cosmetics trays, adding the following allergens: DMDM hydantoin, fragrance mix 2, phenoxyethanol, ethylene-urea-melamine formaldehyde resin, disperse blue mix and methylisothiazolinone 0.2% in aqueous solution (Chemotechnique). The results were + for MI after 48 and 96 hours. The test was considered relevant due to the presence of this allergen in several products used by the patient, such as shampoos, hair conditioners and moisturizing creams. The frequent use of these products explained the appearance of disseminated lesions and for so long a time. The lesions regressed after treating the patient with topical steroids (betamethasone) and instructing her to use alternative products without the causative agent.

**Figure 1:**
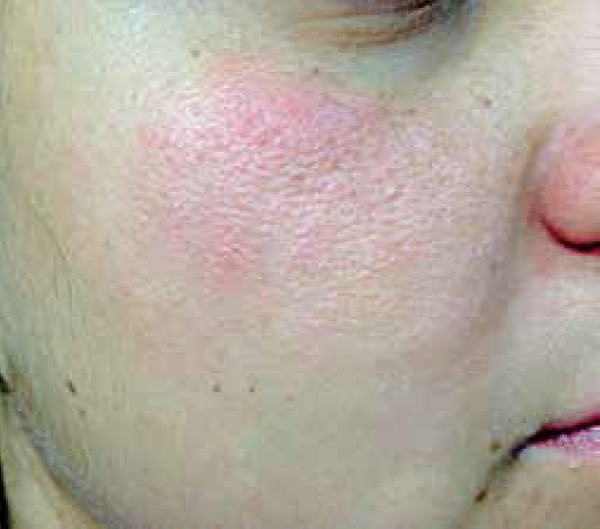
Erythematous, papular, malar plaque

**Figure 2:**
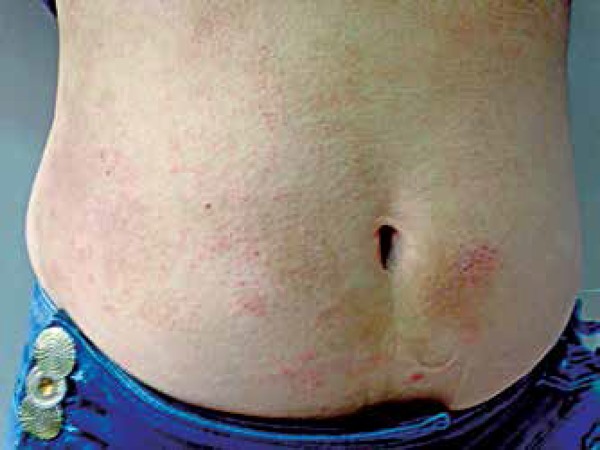
Erythematous-brownish, scaly plaques on the abdomen

In the case reported above, the patient had a widespread rash with prolonged course, which impared her quality of life. The correct diagnosis led her cure, and was only possible because of the patch testing with MI, since the testing with MCI/MI was negative.

MI is an important emerging allergen whose sensitization frequency is rising.

Increased sensitization to MCI/MI in Brazil is a reality, but, unfortunately, this mixture does not allow the detection of all cases of allergy to MI, as seen before. Sensitization to MI should be considered in patients with suspected allergy to cosmetics and/or sunscreens, rash on the face and/or disseminated throughout the body, as illustrated above.^1-5^

We suggest the inclusion of MI in aqueous solution into the Brazilian standard tray, at least temporarily, until international and national norms regulate its use.
